# Ab-Initio Investigations of Magnetic Properties and Induced Half-Metallicity in Ga_1−*x*_Mn*_x_*P (*x* = 0.03, 0.25, 0.5, and 0.75) Alloys

**DOI:** 10.3390/ma10070766

**Published:** 2017-07-07

**Authors:** Amel Laref, Abeer AlMudlej, Slimane Laref, Jun Tao Yang, Yong-Chen Xiong, Shi Jun Luo

**Affiliations:** 1 Department of Physics and Astronomy, College of Science, King Saud University, Riyadh 11451, Saudi Arabia; amodlej@ksu.edu.sa; 2 Department of Physics, National Taiwan University, Taipei 106, Taiwan; 3 Fachbereich Chemie, Philipps-Universität Marburg, Hans-Meerwein-Str., D-35032 Marburg, Germany; laref_s@yahoo.fr; 4 School of Science, Hubei University of Automotive Technology (HUAT), Shiyan 442002, Hubei, China; jtyang@huat.edu.cn (J.T.Y.); xiongyc_lx@huat.edu.cn (Y.-C.X.); sjluo@huat.edu.cn (S.J.L.)

**Keywords:** half-metallic, spintronic, optoelectronic devices, III-V compounds

## Abstract

Ab-initio calculations are performed to examine the electronic structures and magnetic properties of spin-polarized Ga_1−*x*_Mn*_x_*P (*x* = 0.03, 0.25, 0.5, and 0.75) ternary alloys. In order to perceive viable half-metallic (HM) states and unprecedented diluted magnetic semiconductors (DMSs) such as spintronic materials, the full potential linearized augmented plane wave method is utilized within the generalized gradient approximation (GGA). In order to tackle the correlation effects on 3d states of Mn atoms, we also employ the Hubbard U (GGA + U) technique to compute the magnetic properties of an Mn-doped GaP compound. We discuss the emerged global magnetic moments and the robustness of half-metallicity by varying the Mn composition in the GaP compound. Using GGA + U, the results of the density of states demonstrate that the incorporation of Mn develops a half-metallic state in the GaP compound with an engendered band gap at the Fermi level (*E_F_*) in the spin–down state. Accordingly, the half-metallic feature is produced through the hybridization of Mn-d and P-p orbitals. However, the half-metallic character is present at a low *x* composition with the GGA procedure. The produced magnetic state occurs in these materials, which is a consequence of the exchange interactions between the Mn-element and the host GaP system. For the considered alloys, we estimated the X-ray absorption spectra at the K edge of Mn. A thorough clarification of the pre-edge peaks is provided via the results of the theoretical absorption spectra. It is inferred that the valence state of Mn in Ga_1−*x*_Mn*_x_*P alloys is +3. The predicted theoretical determinations surmise that the Mn-incorporated GaP semiconductor could inevitably be employed in spintronic devices.

## 1. Introduction

Diluted magnetic semiconductors (DMSs) have attracted much attention in optoelectronic and spintronic applications. These promising materials represent potential candidates designated to be typical half-metallic ferromagnets with 100% spin polarization at the Fermi level and this behavior is related to the incorporation of a convenient doped atom. Furthermore, the semiconductors substituted by 3d transition metals (TMs) turn salient, owing to the feasibility of merging magnetism and semiconductor features [1,2,3,4,5,6,7,8,9,10,11,12,13,14,15]. The 3d-TM doped III-V compounds behave as III-V DMS, which captivated a tremendous amount of attention for a viable growth in efficient and miniaturized electronic devices [16]. Various DMS materials [7,8,9,10,11,12,13,14,15] have been exhaustively explored from theoretical and experimental perspectives with the purpose of designing powerful devices such as outstanding smart memory chips, super smart diodes, spin valves, and spin field-effect transistors. Accordingly, it is necessary to find an appropriate system designed for fabricating spintronic devices. A good doping level of GaAs leads to the production of volatile memory chips [15]. It is also feasible to utilize gallium nitride (GaN)-doped dilute magnetic semiconducting films in magneto-optical applications. The intended usage is in the form of room temperature ferromagnetic layers for a spin-polarized light emitting diode. Thus, it is essential to dope the host GaN film with manganese for these layers, while still maintaining a good crystallinity and semiconducting features with room temperature ferromagnetic properties. After the revelation of half-metallicity in a semi-Heusler alloy [5], many considerations were emphasized for examining the electronic and magnetic features of various kind of systems in order to discover new half-metallic ferromagnets [2,3,4,5,6,7,8,9,10]. It was ascertained that the most feasible doping percentage of GaAs systems with Mn dopants is approximately 10% or 20% since they exhibit a hysteric response to an applied magnetic field. Subsequently, the researchers paid further attention to investigating the III-V-based DMS and profound endeavors were effectuated by synthesizing ferromagnetic semiconductors over decades [2,3,4,5].

In the recent years, the scrutiny on TM doped III-V DMS was intensified in order to discover novel half-metallic ferromagnetic (HMF) materials with a high Curie temperature (*T_C_*), and to enhance their ferromagnetic characteristics [17,18,19,20,21]. It is compulsory to add a considerable amount of magnetic elements (a few percent or more), surpassing the solubility limit in III-V semiconductors, in order to inspect the magnetic cooperative mechanism in DMSs. In the group of III-V semiconductor compounds, gallium phosphide (GaP) represents an interesting system composed of a cubic zinc blende (ZB) structure and it has broad usages in cellular phones and electronic equipment such as semiconductor lasers and optoelectronic devices, etc. GaP is regarded as a convenient host compound to design DMS material and this could be effectuated through doping by TMs, as corroborated via several theoretical and experimental reports [10,11,12,13,14,15,16,17,18,19,20,21,22,23,24,25,26,27]. Based on AC magnetization measurements, the affirmation of a ferromagnetic state in bulk sintered GaP substituted by 3% Mn is procured at *T_C_* of 600 K, which is substantially larger than the previous examinations. The field location and line width of the resonance exhibited a significant temperature related to ferromagnetic resonance spectroscopy (FMR) spectra. Further validation of high temperature ferromagnetism has been detected via a non-resonant derivative signal centered at zero field at 600 K. Significantly, the occurrence of ferromagnetism is joined to the dilute partition of Mn^2+^ in the sample and not from the clusters of Mn^2+^ or other related impurities [14,24,25,26]. Subsequently, the augmentation in the electron conductivity of the GaP semiconductor could take place by doping with impurities that possess magnetic properties, such as manganese. The occurrence of a ferromagnetic state in these systems would be fruitful for designing novel microelectronics. During the last few years, extensive theoretical and experimental inspections have been concentrated on the magnetic properties of Mn-doped III-V semiconductors. 

It would be valuable to explore gallium manganese phosphate (Ga_1−*x*_Mn*_x_*P) alloys with a specific proportion of manganese atoms owing to their technological applications in electronic devices. First-principle calculations of various III-V-DMS compounds have been carried out using the Korringa Kohn Rostoker-Coherent Potential Approximation (KKR-CPA) method, as reported in Ref. [27]. According to their results, the substitutional Mn in AlP, GaP, and InP systems with a composition of 5% illustrates ferromagnetism at room temperature. Based on both full-potential and pseudopotential schemes, a systematic study of the magnetic features of 3d-TM doped GaAs and GaP materials has previously been carried out [26,28]. Hence, the emergence of the ferromagnetism at room temperature, was identified in GaAs and GaP compounds doped by Cr-, V-, and Mn-elements with a concentration of 25%. Intriguingly, these materials could be prospective candidates for spintronic technology applications. In the past two decades, the origin of ferromagnetism in Mn-doped III-V semiconductors is still being investigated, and it is imperative to scrutinize the related electronic properties. Consequently, the examination, computational modeling, and simulation process are carried out because of the difficulty in the synthesis, growth, and characterization of Mn-doped III-V semiconductors. The prediction of numerous attitudes of systems is facilitated by the usage of computational techniques and recent advanced ab-initio calculations. The computational methods have already been employed for various systems by means of the first principle calculations and the theoretical works were consistent with the experimental results. Thus, we are motivated to verify the magnetic response and half-metallic character based on the density functional theory (DFT) approximation. 

The experimental measurement of TM-doped GaP [26] triggered our motivation to analyze the magnetic response and half-metallic character. Furthermore, the solubility limit of TMs in III-V based DMS materials was less than 4% [26]. Hence, it is essential that the dilute limit of the Mn-doped GaP system is less than 4% for producing credible data and to support the experimental works [26]. Accordingly, we report a theoretical investigation regarding the electronic structure and magnetic properties of Mn-doped GaP with a doping concentration of *x* = 0.03, in addition to higher concentrations *x* = 0.25, 0.5, and 0.75. The other goal of current scrutiny is to inspect the change of half-metallicity with the variation of composition until the dilute limit. The examination of magnetic origin in semiconductors with nonmagnetic atoms relies on p-d hybridization, which is the main contributor to HMF. In this work, the examination of the electronic and magnetic features of Ga_1−*x*_Mn*_x_*P (*x* = 0.03, 0.25, 0.50, 0.75) alloys is handled by employing DFT [29] in the framework of the all-electron full potential linear augmented plane wave (FP-LAPW) method. The latter is embodied in the WIEN2k code [30], which has been demonstrated to be one of the most exact techniques for computing the electronic structure of materials. Accordingly, we applied the state-of-the-art generalized gradient approximation (GGA) combined with Hubbard U approach, and the on-site Coulomb interactions approach [31,32,33,34] was implemented in the FP-LAPW plus a local orbital method (lo) [30]. We computed the electronic density of states and magnetic characteristics of Ga_1−*x*_Mn*_x_*P alloys using the FP-LAPW + lo technique [30] with both GGA and GGA + U techniques. We thoroughly analyzed the results and predicted the magnetic properties, which are based on the inspection of the density of state results and magnetic moments. 

The eventual usage of electron spin within the electronic charge in contemporary devices has resulted in a prominent concern regarding DMS materials. The straightforward knowledge regarding the electronic states of a specific atom would be attainable through X-ray absorption spectra (XAS). Over the past few years, theoretical reports have been carried out on (Ga,Mn)As DMSs to elucidate the measured XAS [35,36,37,38,39]. Therefore, we aim to perform ab-initio calculations to examine the electronic structural characteristics of the Mn K-edge X-ray absorption spectrum in Mn-doped GaP material. In the present work, the X-ray absorption near-edge structure (XANES) spectra of Mn- in Ga_1−*x*_Mn*_x_*P alloys will be elucidated, which could be compared to other half-metallic systems. Subsequently, to reveal this behavior and to shed light on the mechanism of the prominent magnetic properties of Ga_1−*x*_Mn*_x_*P alloys, we analyze the X-ray absorption near edge structure (XANES) features for the Mn K-edge spectra. This scrutiny provides the first theoretical indication of XANES spectra at the Mn K-absorption edge in Ga_1−*x*_Mn*_x_*P alloys. This finding has been ascertained in previous experimental measurements of the Mn K-edge in GaMnAs.

The paper is organized as follows. The computational details of the employed method will be briefly portrayed in the following section. Using GGA and GGA + U techniques, the magnetic and electronic aspects, essentially the density of states (DOS), will be discussed in Section 3. The resulting magnetic properties and the Mn K-edge X-ray absorption spectra of Ga_1−*x*_Mn*_x_*P alloys are also reported with the GGA + U approach. We will end with the conclusions regarding the electronic structure and magnetism of Ga_1−*x*_Mn*_x_*P alloys.

## 2. Method of Simulation

In the framework of the FP-LAPW + lo methodology, the self-consistent calculations of electronic structures of Mn-doped GaP systems are performed, as embodied in the WIEN2K code [30]. Using the FP-LAPW scheme, the core states are handled in a full relativistic manner, whereas the semi-core states and valence states are considered in a semi-relativistic way (by omitting the spin orbit effect). Every atom is enclosed in a muffin-tin sphere, and the two regions are separated by the entire space. The first region is composed of inner non-overlapping spheres, although the interstitial region is occupied by the remaining space. The selection of non-overlapped muffin-tin radii (RMT) of Ga, Mn, and P should be quite large to procure almost adjoining spheres and guarantee the reduced interstitial space. Therefore, the radii of the muffin-tin spheres are 2.45a_0_ for Ga and Mn, and 2.05a_0_ for P, where a_0_ represents the Bohr radius. The generation of the exchange and correlation (XC) potentials are based on the GGA technique within the parameterization of Perdew-Burke-Ernzerhof (PBE), as referred to in Ref. [31]. The expansion of potentials is represented by the spherical harmonics of *l* maximum value that is up to *l_max_* = 10, while the expansion of the wave functions of valence electrons are occurring inside the muffin-tin spheres. The expansion of plane waves or Fourier series takes place inside the interstitial regions, albeit the plane wave cutoff value of *R_MT_*. *K_max_* is extending with a limitation value of eight (*R_MT_* characterizes the muffin-tin sphere radii and *K_max_* represents the biggest plane wave vector cutoff of the basis set in which *K_max_* controls the exactitude of the calculation). A convergence test was performed for the well converged results and *G_max_* =14 a.u.^−1^ was set-up for the Fourier expansion of potential inside the interstitial part. 

On the basis of the DFT scheme, the presentation of the electronic structures of Mn-TM with a 3d state are specious owing to their strong correlation effects. The strongly correlated behaviour of TMs has impeded the quantified modification with ab-initio calculations utilizing the local spin density approximation (LSDA) and spin polarized GGA procedures. The major issue associated with these techniques [31,32] is attributed to the correct determination of the electronic structure of the material. However, the GGA + U approach [33,34] was observed to accurately reproduce the measured data and illustrates the importance of correlation effects. It was demonstrated that DFT + Hubbard U approximation is an effective method to tackle the static correlation effects in materials with transition metals [33,34]. The precise treatment of the correlation effect based on many body theories is very often in conjunction with the applied models (as in, the Hubbard model). By the virtue of the Hubbard model, the tackled orbitals are characterized as an orbital-depending potential together with on-site Coulomb and exchange interactions, U and J. Thus, the double counting inside the non-spherical part of the potential should be prevented by employing U_eff_ = U − J in place of U [33,34], although we should exclude the multipolar terms that correspond to J in the extra GGA + U potential. Thus, the choice of an appropriate U value is acquired by applying several methodologies. These techniques may employ the experimental data based on self-consistent schemes or X-ray photoelectron spectroscopy (XPS) [33,34,35,36]. Consequently, it was earlier determined via XPS spectrum that the d-d Coulomb correlation of Mn values varies from 3.0 to 5.5 eV [36] and this essentially depends upon the structural data of the samples. The evaluated value of the effective Hubbard U parameter of the Mn 3d states was approximately 5.0 eV. The calculations rely upon the supercell technique and Ga atom (cation site) belonging to the supercells of 1 × 1 × 1 and 2 × 2 × 2 of GaP is substituted with the dopant Mn-element. Subsequently, the construction of these supercells has been set to achieve 3.125%, 25%, 50%, and 75% doping with Mn, respectively. In this work, we consider the ferromagnetic case. The convergence of self-consistent calculations is attained by monitoring the total energy tolerance of 10^−4^ Ry and converged charge of 0.0001e. We applied the modified tetrahedron technique [30] within 4000 k-points in the irreducible Brillouin zone (IBZ) to assure a high precision for our results.

## 3. Results and Discussion

**Figure 1:**
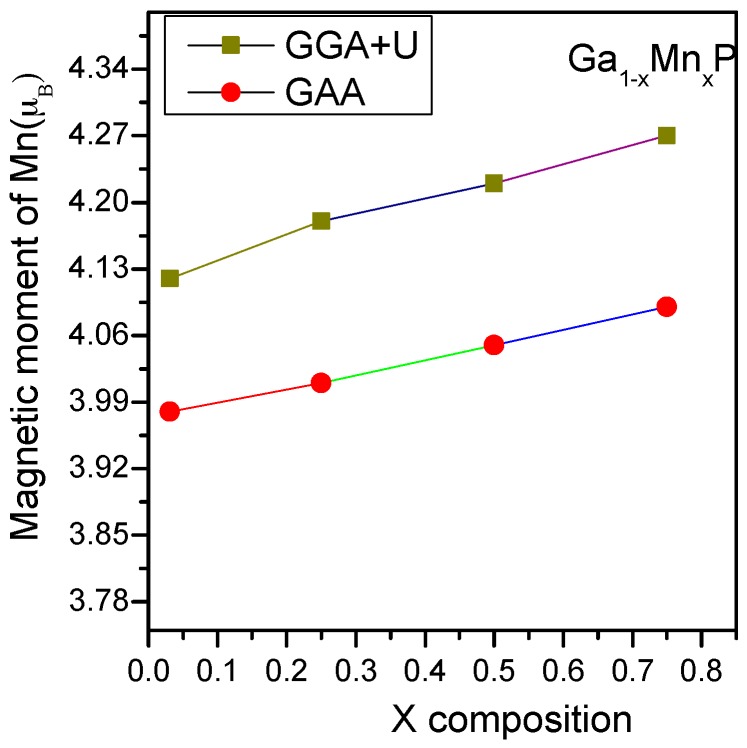
Atom resolved magnetic moments of Mn-doped Ga_1−*x*_Mn*_x_*P (*x* = 0.75, 0.5, 0.25, 0.03) alloys versus the variation of composition *x*.

Using both GGA and GGA + U electronic structure calculations, the lattice parameter of the supercell was optimized in order to compute the electronic and magnetic properties of Ga_1−*x*_Mn*_x_*P alloys with *x* = 0.03, 0.25, 0.5, and 0.75. The spin-polarized calculations are handled to evaluate the on-site magnetic moment at the Mn sites for all ferromagnetic structures. For all the studied alloys, the local magnetic moments on the Mn atoms of ferromagnetic states are depicted in Figure 1. It is remarkable that the results of local moments on Mn atoms determined by GGA + U are slightly higher than those computed with the GGA technique. This is mainly owing to the inclusion of the d-d Coulomb correlation in the Mn atom when using the GGA + U technique. It is evident that a global magnetic moment of approximately 4µ_B_ is acquired per supercell for all the systems under investigation, which is in gratifying conformity with previous works [24,25,26,27]. As anticipated, the Mn-doping element represents the major contribution to the global magnetic moment with the change of doping concentration. From Figure 1, it is evident that by varying the composition of Mn in the GaP system, the localized magnetic moment (GGA + U) displays a small variation in the range of 4.10 µ_B_ or 4.26 µ_B_ owing to the larger volume of Ga compared to Mn. This could probably lead to the preference of ferromagnetic order of the manganese atom in the GaP system. In all the studied alloys, the phosphorous neighbors of Mn together induce an insignificant contribution between −0.05 µ_B_ and −0.09 µ_B_, although the Ga second neighbors of Mn together contribute very weakly in the range of 0.04 µ_B_–0.07 µ_B_ to the magnetic moment. In the case of the substitution of a tetravalent Mn-atom instead of a trivalent Ga element, three electrons are considered to set a bonding with P elements and the rest of the electrons are located in the additional states at *E_F_*, which develops magnetic states in the concerned alloys. The Mn-3d states represent the major cause of magnetism in Ga_1−*x*_Mn*_x_*P (*x* = 0.03, 0.25, 0.5, and 0.75) alloys, apart from the p-d hybridization. Moreover, the magnetic behavior of these alloys may be associated with the partially filled t_2g_ orbitals of Mn-3d states [24,25,26,27]. However, the emerged magnetic moment on the Ga-/P-element is negligibly insignificant, aligning parallel/antiparallel to the Mn-element, respectively. The present findings assume that the developed magnetism is owing to the exchange interaction between Mn-atoms and the host material. It is also essential to emphasize that this behavior could significantly contribute to determining the inspected spintronic effect [1,2,3,4].

**Figure 2:**
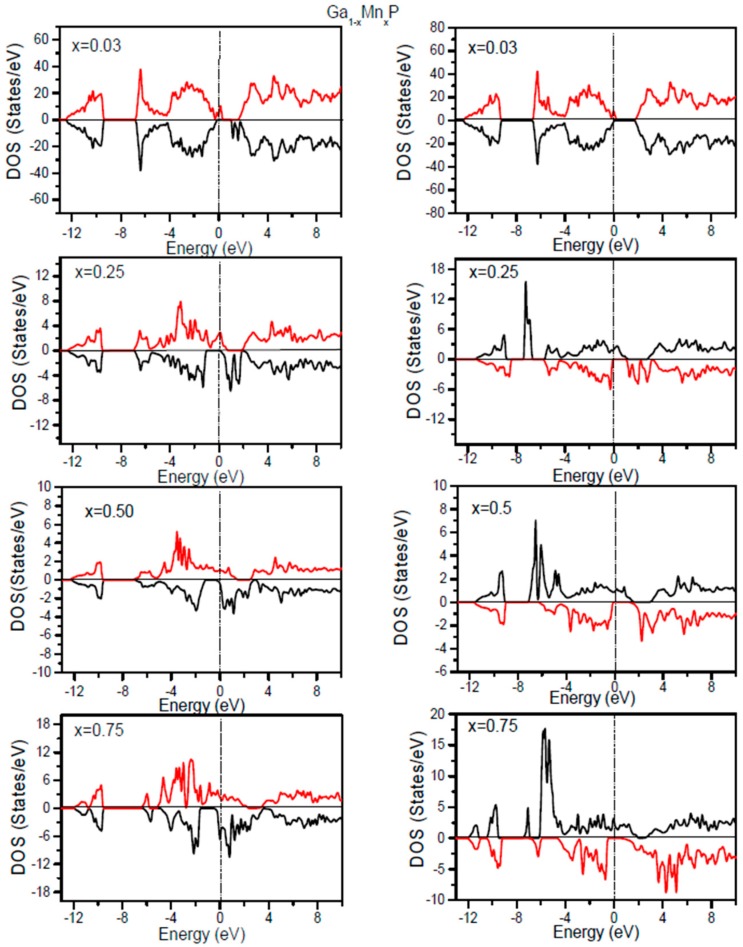
Computed total density of states of Ga_1−*x*_Mn*_x_*P alloys (*x* = 0.03, 0.25, 0.5, and 0.75) for spin up and spin down. The vertical dash line denotes the Fermi level. DOS for majority and minority spin channels are illustrated by the +eV and −eV value, respectively. The left panel displays the GGA results and the right panel indicates the GGA + U results.

For the comprehension of the electronic structures of Ga_1−*x*_Mn*_x_*P alloys, it is informative to scrutinize the associated densities of states. By means of the GGA + U approach, the Coulomb interaction effect of the correlation of 3d electrons could be evinced in the total densities of states (TDOS). It is compulsory to discern the magnetic nature of the considered alloys. On the basis of optimized lattice constants, the spin polarized electronic density of states and robustness of half-metallic aspects are computed. In order to acquire a profound vision into the variation of the electronic structure of the ferromagnetic Ga_1−*x*_Mn*_x_*P (*x* = 0.03, 0.25, 0.5, 0.75) alloys, we calculate the total TDOS using the GGA and GGA + U approaches, as plotted in Figure 2. Based on the aid of the DOS, it is essential to comprehend the source of states of Ga_1−*x*_Mn*_x_*P alloys. Evidently, the more electrons are located in the spin-up (↑) state comparatively to the spin-down (↓) state. The occurrence of a band gap around *E_F_* for the minority-spin channel of Ga_1−*x*_Mn*_x_*P (*x* = 0.03, 0.25, 0.5, 0.75) has been revealed in the resulting GGA + U DOS, whereas some valence states cross *E_F_* for the spin-up state and relocate to the conduction states. Subsequently, these promising materials are predicted to possess a HM aspect. The TDOS of the considered alloys at low doping Mn compositions illustrates an energy gap in the spin-down state owing to the splitting of DOS at *E_F_*, which consequently conducts to HM systems. Note that the binary GaP compound has a semiconducting nature possessing a band gap of 2.32 eV [14,26,27]; whereas its corresponding features are drastically altered by doping with an Mn-atom. It is observed that the incorporation of manganese yields supplementary states in the uppermost section of the valence states for the majority-spin and the downward region of valence states for the minority-spin component, thus leaving a band gap. Therefore, the addition of Mn atoms in the system maintains the semiconductor aspect of GaP for the spin-down state. The metallic character in the spin-up states and semiconductor nature in spin-down states yield Ga_1−*x*_Mn*_x_*P ternary alloys as realistic HM ferromagnets. It is evident that the TDOS computed with the GGA + U indicates an HM state for the minority spin channel at various *x* compositions, whereas the states near *E_F_* display splitting and the HM gap are increased in comparison with the TDOS results calculated by means of the GGA. This situation is attributed to the correlation effects, which are important in 3d states of Mn. Note that the HM-behavior is present at a low composition of Mn in the GaP compound, whereas the increase of Mn content leads to a metallic state with GGA. Owing to the emergence of this specific mechanism, these prosperous materials are practical for producing a thoroughly spin polarized current. Moreover, they are reliable for maximizing the efficiency of spintronic devices.

**Figure 3:**
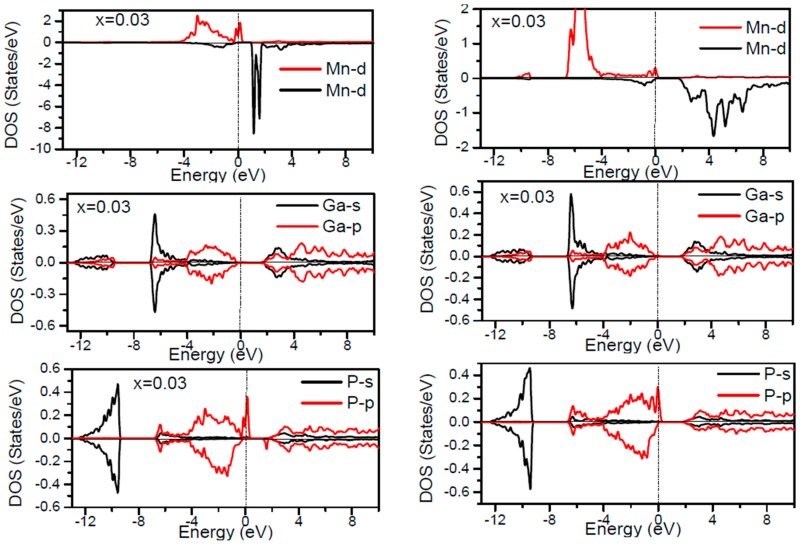
Computed partial DOS of spin polarized contributions from Ga-s,p, As-s,p, and Mn-d states in the Ga_1−*x*_MnP (*x* = 0.03) alloys for spin up and spin down. The left panel indicates the GGA calculations and right panel denotes the GGA + U calculations. The vertical dash line denotes the Fermi level.

**Figure 4:**
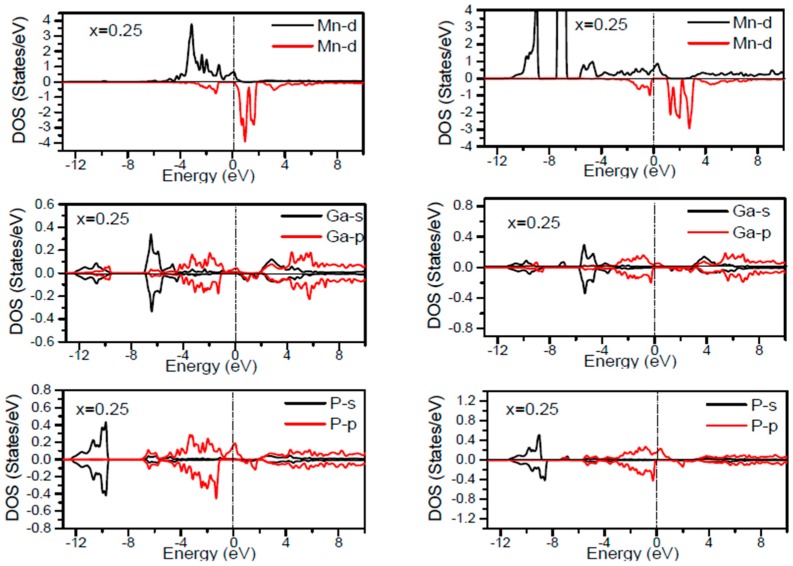
Computed partial DOS of spin polarized contributions from Ga-s,p, As-s,p, and Mn-d states in the Ga_1−*x*_MnP (*x* = 0.25) alloys for spin up and spin down. The left panel indicates the GGA calculations and right panel denotes the GGA + U calculations. The vertical dash line denotes the Fermi level.

**Figure 5:**
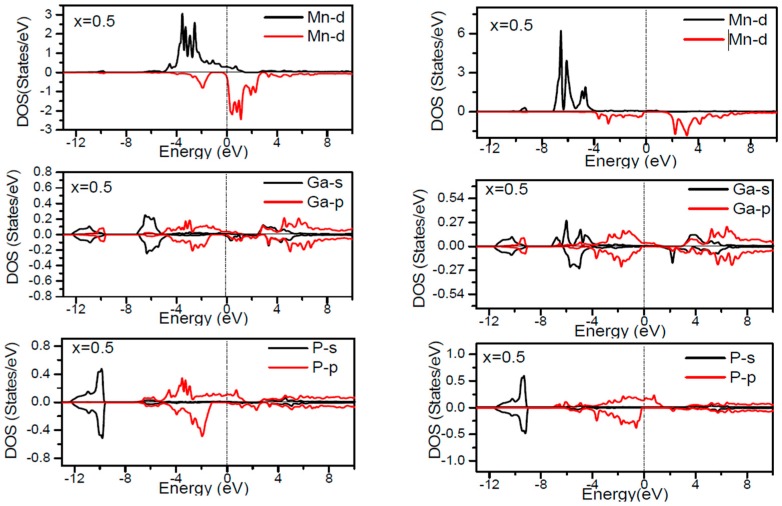
Computed partial DOS of spin polarized contributions from Ga-s,p, As-s,p, and Mn-d states in the Ga_1−*x*_MnP (*x* = 0.5) alloys for spin up and spin down. The left panel indicates the GGA calculations and right panel denotes the GGA + U calculations. The vertical dash line denotes the Fermi level.

**Figure 6:**
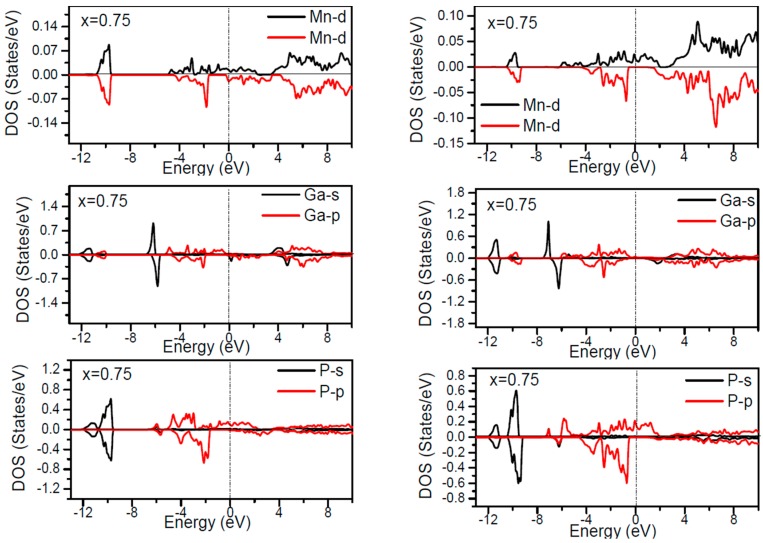
Computed partial DOS of spin polarized contributions from Ga-s,p, As-s,p, and Mn-d states in the Ga_1−*x*_MnP (*x* = 0.75) alloys for spin up and spin down. The left panel indicates the GGA calculations and right panel denotes the GGA + U calculations. The vertical dash line denotes the Fermi level.

**Figure 7:**
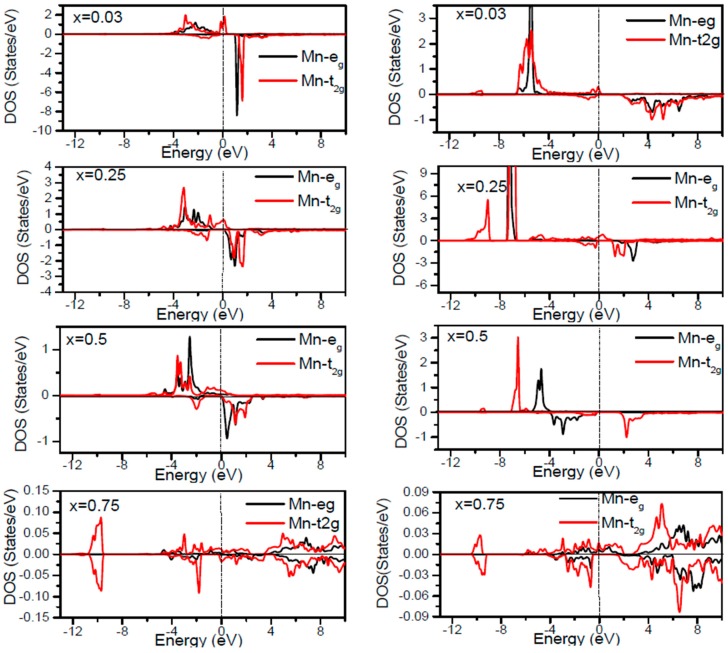
Computed t_2g_ and e_g_-DOS of the Mn atom in the Ga_1−*x*_Mn*_x_*P (*x* = 0.03, 0.25, 0.5, and 0.75) alloys for spin up and spin down. The left panel shows the GGA results and right panel denotes GGA + U results. The vertical dash line denotes the Fermi level.

In order to inspect the assistance of different states responsible for the HM character of these systems near *E_F_*, the spin dependent partial DOS (PDOS) of Ga_1−*x*_Mn*_x_*P (*x* = 0.03, 0.25, 0.5 and 0.75) materials are depicted in Figure 3, Figure 4, Figure 5, Figure 6 and Figure 7 by employing the GGA and GGA + U approaches. It is evident that the states in the locality of *E_F_* are predominantly due to Mn-3d and P-p levels, with an insignificant participation from the Ga-p levels for the majority spin component (as seen in Figure 3, Figure 4, Figure 5, Figure 6 and Figure 7). In the spin-up states, the bands at *E_F_* illustrate a mixture of Mn-d and P-p like states, which play a valuable role for implying the magnetism at all the various compositions. From the computed DOS, the P-s and Mn 4s states are repelling each other. The P-s state is moved close to the core and Mn-4s into *E_F_*. As is apparent, the uppermost valence band (VB) and lower conduction band (CB) of the minority spin components show that the Mn-d and P-p bands are the most responsible to determine the energy band gap at *E_F_*. Note that the contribution of Ga-s, Ga-p, and P-s bands is mostly insignificant for both spin components. The PDOS computed with GGA + U illustrates a HM state for the minority spin channel at various *x* compositions. The states close to *E_F_* are also split and the HM gap is increased compared to the results produced with the GGA. Subsequently, by increasing the Mn- composition from 0.25 to 0.75, the spin-up d-states split and their effective assistance at *E_F_* increases. By the virtue of the theory of crystal field splitting, the emerged HM ferromagnetism in this striking material is assessed through the splitting of P ions. In fact, this would drive to the conversion of 5-fold degenerated Mn-3d orbitals into 3-fold degenerated t_2g_ (d_xy_, d_zx_, d_yz_), states and doubly degenerated e_g_ (d_z2_, d_x2−y2_) states, as clearly noticed in Figure 7.

According to the DOS results, there are hybridized Mn-3d levels with a P-3p neighbor level of Mn in the case of Mn-doped GaP materials. It is evident that the DOS ranging between −5.5 and 1.5 eV is essentially reflected by the Mn-d and P-p orbitals, with a relatively insignificant participation of Ga-p levels at both spin states (see Figure 3, Figure 4, Figure 5, Figure 6 and Figure 7). The assistance of Mn-3d states in the spin-up states is separated into two sections: first, the states emerging through the Mn-3d (e_g_-doubly degenerate orbitals) states lie at approximately −1.81 eV, and second, the location of Mn 3d t_2g_-triply degenerate states, roughly at −0.46 eV, is positioned upward of e_g_ states (see Figure 7). Subsequently, these states exhibit a strong hybridization of wave functions for t_2g_ state and weak hybridization for e_g_ states, with P-p states leading to the occurrence of bonding and anti-bonding hybrids. Thus, this hybrid conducts to a shift in the valence states of the concerned alloys to higher energies. The extra Mn doped GaP displays a significant repulsion between bonding and anti-bonding states. The hybridization between these states essentially contributes to the determination of ferromagnetic behavior in these materials for various dopant compositions *x*. Owing to the considerable p-d hybridization, the partially occupied Mn-t_2g_ antibonding orbitals participate in the formation of the band gap. This latter is subject to the splitting of the two symmetry states in the spin-down component and thereby the half-metallic gap occurs. However, in these alloys, the net magnetic moment and HM behavior are characterized by the partial occupancy of spin-down state at *E_F_*. As demonstrated in previous research [24,25,26,27], the d-orbitals of the Mn atom are majorly positioned in the band gap, and they may be separated via the crystal field and exchange interaction. The crystal field produced via the four P nearest-neighbors yields the splitting of Mn-d levels into doubly degenerated e_g_ states and triply degenerated t_2g_ states (Figure 7). Accordingly, the substantial interaction between the 3d-t_2g_ orbitals of Mn and P-3p orbitals leads to the pushing up of the 3d-t_2g_ states upward of the e_g_ states [15,16,17,24,25,26,27]. The computed GGA + U band gap of minority spin varies between 1.7 and 0.8 eV when varying the *x* concentration. However, for a high Mn composition, the compound shows a high spin polarized state within the GGA technique. It is concluded that the studied alloys own ferromagnetic behaviors, which are in good accordance with the previous finding reported experimentally [14,24,36].

Moreover, the presence of a half-metallic gap, which indicates a minimal gap for spin excitation in the minority-spin channel, is an impressive characteristic of these promising systems. To understand the HM nature of these materials, the minimum value of (*E_F_* − E^v^_top_) or (E^c^_tot_ − *E_F_*) should be computed, where E^v^_top_ and E^c^_tot_ denote the energy associated with the uppermost valence states and downward conduction states, respectively. In different way, the control of realistic HM ferromagnet is attributed to a non-zero HM gap relatively to the band gap in any spin channel [12,15,17,24,25,26,27]. On the basis of this deliberation, it is surmised that the augmentation of *x* content in Ga_1−*x*_Mn*_x_*P alloys from 0.03 to 0.75 leads to the half-metallic nature in these corresponding systems by using the GGA + U approach. However, the presence of a HM gap is found at a low content of Mn when using the GGA approach. Notably, our results illustrate half-metallicity in Ga_0.50_Mn_0.50_P and Ga_0.25_Mn_0.75_P alloys by using the GGA + U approach, whereas the previous studies found a metallic state in these systems by using the LSDA approach, which confirms our results [24,25,26]. Our GGA energy gaps are smaller than those obtained with the GGA + U approach since the d-d Coulomb correlation is important for the 3d-state of Mn. The resulting band gap is approximately 1.7 eV for the dilute limit of the magnetic compound (*x* = 0.003). It is evident that doping with TM leads to the diminishing of the band gap to a lesser extent than that of the pure GaP compound, i.e., 2.3 eV [26]. Within the GGA + U approach, a reduced band gap has been detected at approximately 0.8 eV in the minority spin component for high-Mn content doping in GaP, as displayed in Table 1. The HM gap decreases as the Mn-composition augments in the GaP compound. This situation is attributed to the localized character of Mn-d orbitals at *E_F_*, which reduces with the augmentation of the doped composition from 0.03 to 0.75. The Mn-d orbitals are significantly hybridized with P-p orbitals for the various doping levels (*x* = 0.25, 0.5, 0.75), arising in an energy gap for the spin-down state with the greater distribution between bonding (e_g_) and antibonding (t_2g_) orbitals. Significantly, the half-metallicity was determined to remain intact as the dopant concentration varies between 0.03 and 0.75. 

A deep knowledge about optical spectroscopy is important to understand the underlying properties of materials. In this respect, the incorporation of not only the vacant and filled levels of the electronic structure, but also the band character, is required to attain the optical properties. The XAS constitutes a realistic experimental tool for analyzing the valence states of a particular element [35,36]. The relation of the XAS intensity (*I*) of a peculiar atom (*A*) in the dipole approach is denoted as follows:

*I* ∝ ν^3^|<Ψ_val_|*r*|Ψ_core_>|^2^*N^A^_l_* (*h*ν − E_core_) (1)

where the photon frequency is indicated by ν and *N^A^_l_* represents the *l*-like partially non-occupied DOS of atom *A*. Notably, *A* denotes Mn and *l* is related to Mn-states. Here, the contribution of Mn 4p states is accompanied by the Mn K-edge spectra. This is mainly attributed to the angular momentum selection rule by means of the dipole approach. In the light of experimental techniques such as XAS and resonant X-ray scattering, we can procure a straightforward guideline of the occupied orbitals. Thus, the non-occupied DOS in the vicinity of the Mn K-edge grants a supplied elucidation regarding the incorporation of the orbitals into the chemical bonding. In order to acquire further comprehension by means of the Mn K-edge XAS spectra of Ga_1−*x*_Mn*_x_*P ternary alloys, our calculations of XAS spectra are procured using the GGA + U approach. The valence state of Mn was investigated by means of XANES at the K-edge of Mn. Note that the XANES is sensitive to the valence state of the absorber. Accordingly, XAS has been performed with Mn-doped GaP material to identify the electronic environment of Mn^3+^. 

**Figure 8:**
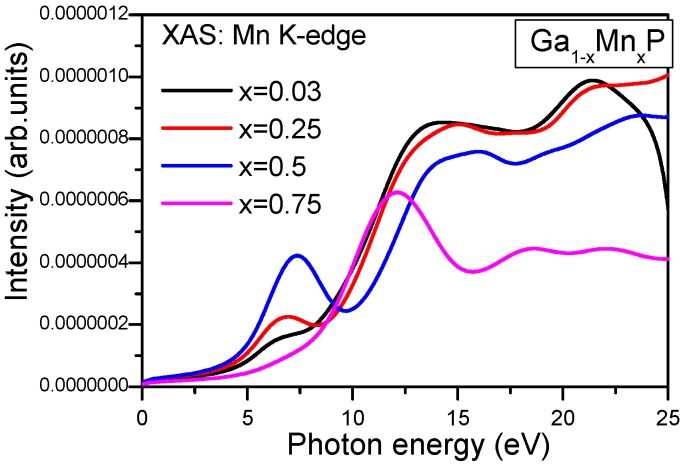
K edge X-ray absorption spectrum of Mn in Ga_1−*x*_Mn*_x_*P alloys at different concentrations of *x* = 0.03, 0.25, 0.5, and 0.75 within the GGA + U approach.

In Figure 8, we illustrate the edge part of the Mn K-edge X-ray absorption spectra of Ga_1−*x*_Mn*_x_*P (*x* = 0.03, 0.25, 0.5, and 0.75) alloys. At first glance, the plots display some similar features in the XANES spectra of Ga_0.75_Mn_0.25_P and Ga_0.5_Mn_0.5_P alloys, which implies a strong effect of p states (see Figure 8). By considering the aforementioned results, assisted by the computed total and partial (i.e., referred to Mn 3d orbitals) DOS of Ga_1−*x*_Mn*_x_*P systems, a qualitative similarity between the main peaks of Ga_0.75_Mn_0.25_P and Ga_0.50_Mn_0.50_P emerges, lying above the energy of the K-absorption threshold. According to the dipole selection rule, the allowed transition to bands contain only p states. For this reason, two well resolved pre-edge peaks are developed at 5.0 and 16 eV (see Figure 8). The Mn K-edge X-ray spectra in Ga_1−*x*_Mn*_x_*P alloys illustrate that the pre-edge structures are shifted upward in the energy to elevated photon energies and in intensity versus the change of *x* concentration (Figure 8). The calculated spectra describe a clear splitting in the first two peaks for low Mn-content in GaP materials compared to the relative intensities of XAS of the Ga_0.25_Mn_0.75_P alloy. The first small pre-peak is positioned at approximately 6.9 eV for *x* = 0.03 and 0.25. It is therefore shifted and doubled in intensity for the Ga_0.5_Mn_0.5_P alloy located at approximately 7.3 eV. It is well noticeable that the pre-edge structure contains a marked single peak at approximately 12.6 eV for *x* = 0.03 and 0.25 and is often characterized by weakly permitted dipole transitions from Mn(1s) to Mn(3d/4p) orbitals. Further, the alteration originating from the core hole effect would be dissimilar for the d-orbitals of the pre-edge doublet and the p-orbitals in the conduction states [36,37,38,39,40]. However, the second peak is positioned near 18.6 eV, which is much weaker in the Ga_0.25_Mn_0.75_P alloy compared to the case of low Mn-content in GaP alloys and subsequently, the intensity of the absorption spectrum is decreased above 20 eV. Notably, the second peak of XAS for low content of Mn is more remarkable around 15.1 eV, which is increased in the intensity of absorption beyond 20 eV. As apparent, in the CB, the Mn -4p states are reflected by the absorption lines at an elevated energy. Otherwise, the second structural peak of the Mn K-edge spectra of the Ga_0.75_Mn_0.25_P and Ga_0.50_Mn_0.50_P systems illustrates a wide top spreading from 15 to 20 eV (Figure 8). Nevertheless, its pattern proposes the presence of two components and partially overlapped peaks. 

A remarkable feature about the increase of the intensity of the Mn K-edge spectrum is due to the augmentation of Mn content in GaP. The first pre-edge in XAS signifies that the 3d orbitals (majority-spin channel) are not thoroughly occupied. From the interpretation of XANES, the excited electrons arise from the vacant states upwards of *E_F_* and are produced from the core states. The tetrahedral coordination of ligands (nearest neighbor P ions) enables p-d hybridized states of Mn. In other words, the major K-edge absorption begins and expands into various Mn elements over the section of highly delocalized 4p states. The assignment of p-d hybridized states is positioned at some specific peaks, and this is due to the population of d-electrons in the Mn sphere (4.49 e) and the resulting global magnetic moment (4.0 µ_B_/cell) in all alloys. This population value of d-electrons points to the 3+ valence state of Mn (d^4^ configuration). It is compelling to compare the current K-edge spectra, in which we infer that the 3+ valence states of Mn exhibit a tetrahedral arrangement of P ligands in the ZB-type lattice. Hence, the first pre-peak is present in the spectra with low dopants. Consequently, the pattern of the XANES spectrum is related to the variation of Mn concentration, implying the addition of more valence states of Mn atoms in the GaP system. It is inferred that a peak in the pre-edge structure corresponds to the 3+ valence state of Mn. Notably, this feature was also detected in GaMnAs systems [35,36,37,38,39,40,41], in accordance with the overall comprehension that Mn behaves as an acceptor in Ga_1−*x*_Mn*_x_*P alloys, from which it possesses d^4^ configuration.

In overall, the pre-edge structure for the Mn K-edge of these studied alloys contains one or two insignificant spectral peaks, which possess Mn-3d like orbitals. Notably, these striking characteristics are detected in XANES spectral features for various TM materials [2,35,37,38,39]. With the modification of the Mn-content in the related alloys, a translation shifts and an additional structure is present. Exclusively, for all the studied alloys, the difference appears in the peak pre-edge structure, which is principally assigned to the Mn-3d/4p orbitals. Hence, the quantitative inspection of XANES is related to the alteration of the Mn K-edge threshold energy and the magnitude of pertinent cation–anion charge transfer in these alloys. It is proposed that the bonding and p-d exchange between Mn and P in these alloys are dramatically similar because the XAS line-shapes are predominantly influenced by the hybridization of the t_2g_-symmetric Mn 3d states with the neighboring anion p states. The calculated spectra are consistent with the earlier measurements on GaMnAs alloys [35]. From the previous experiments based on a detailed analysis of X-ray diffraction and X-ray linear dichroism, it was found that Mn atoms occupy Ga sites with a significant +3 valence state [2,35,36,37,38,39,40,41]. It is anticipated from our theoretical realizations that more investigations about the magnetic properties of Ga_1−*x*_Mn*_x_*P alloys will be proposed. From the calculations of the XAS spectra, the Mn-dopant in the GaP compound provides a realistic mapping for interpreting the electronic structure and magnetic features of Ga_1−*x*_Mn*_x_*P systems. 

## 4. Conclusions

In summary, Mn-doped GaP materials are investigated using FP-LAPW methodology with the GGA and GGA + U approaches. The electronic and magnetic features of the Mn-doped GaP compound are explored for various doping compositions, i.e., *x* variation between 0.03 and 0.75 in order to seek half-metallic compounds, which are fruitful for spintronic applications. It was determined with the GGA approach that the computed DOS leads to the HM state for a low composition and to the spin-polarized state for a high content of Mn. It has been demonstrated that the computed GGA + U DOS of Ga_1−*x*_Mn*_x_*P ternary compounds yields a ferromagnetic behavior with the HM gap at *E_F_* in the spin-down component at various compositions *x* between 0.75 and 0.25, in addition to the dilute limit of the compound (*x* = 0.03). It was determined that the Mn-dopant leads to a metallic character in the spin-up component for all the investigated alloys, which are characterized by half-metallic ferromagnets. The half-metallicity remains intact by modifying the Mn-dopant composition in the GaP compound with the GGA + U approach. The half-metallic gap at *E_F_* at the spin-down state slightly decreases as the Mn-content in the GaP system is augmented. The half-metallic state is produced through the hybridized Mn-d and P-p orbitals. The hybridization between the Mn-d and P-p orbitals leads to the pushing of the symmetric d-levels at *E_F_* in the majority spin channel and beyond *E_F_* in the minority spin channel and thereafter, developing a band gap in our ternary alloys at all dopant compositions. This aspect renders these ternary compounds relevant for realistic spintronic devices and assists the demand of their growth experimentally. The Mn-element represents the major contributor to the global magnetic moment and the driven magnetism is due to the existence of Mn-d states at *E_F_* and the exchange interaction between the Mn-element and the host-GaP material. For all doped compositions, the insignificant magnetic moment on the non-magnetic atoms is associated with the p–d hybridized orbitals arising from the Mn-d and P-p states. A first principle prediction of the Mn K-edge X-ray absorption spectrum of Ga_1−*x*_Mn*_x_*P ternary alloys was also represented. Furthermore, the electronic structures of Ga_1−*x*_Mn*_x_*P alloys have been investigated by computing the Mn X-ray absorption near-edge structure spectra. Similarities in the shapes of Mn K-edge XANES spectra of Ga_0.5_Mn_0.5_P and Ga_0.75_Mn_0.25_P alloys are appreciable. The computed K-edge X-ray absorption spectra of Mn in Ga_1−*x*_Mn*_x_*P alloys reveals the +3 valence state of Mn and the occurrence of marked pre-edge peaks at *x* = 0.03 and 0.25. The XANES spectra of Ga_1−*x*_Mn*_x_*P ternary compounds have exhibited a shift in the magnitude of the Mn K-edge absorption threshold as a function of the variation of Mn composition in GaP material. This could enable us to discern the valence states of Mn in Ga_1−*x*_Mn*_x_*P systems. These promising materials could be very beneficial for maximizing their effective applications in spintronic devices. 

## Figures and Tables

**Table 1:** The calculated half-metallic gap of Ga_1−*x*_Mn*_x_*P alloys (*x* = 0.03, 0.25, 0.5, and 0.75) for spin-down channel by using GGA and GGA+U procedures.

Ga_1−*x*_Mn*_x_*P		
	GGA	GGA + U
*x* = 0.03	0.9	1.7
*x* = 0.25	0.2	1.1
*x* = 0.5	0	1.1
*x* = 0.75	-	0.8
